# Programmable and tunable flat-top supercontinuum laser sources via electro-optic intensity and phase modulation scheme

**DOI:** 10.1038/s41598-022-22463-y

**Published:** 2022-10-27

**Authors:** Minhyup Song, Minje Song, Seungyoung Lim, Hyunjong Choi, Taehyun Lee, Gyudong Choi, Youngjin Jung, Joon Tae Ahn

**Affiliations:** 1 Photonic/Wireless Devices Research Division, Electronics and Telecommunication Research Institute, Daejeon, 34129 South Korea; 2 School of Electronics Engineering, Kyungpook National University, Daegu, 41566 South Korea; 3 School of Computer and Information Technology, Korea University, Seoul, 02841 South Korea; 4 School of Electrical Engineering, Korea University, Seoul, 02841 South Korea

**Keywords:** Frequency combs, Fibre optics and optical communications, Optoelectronic devices and components

## Abstract

In this study, we presented flat-topped coherent supercontinuum lasers with tunable repetition rates and programmable spectral bandwidths. Supercontinuum sources with ultra-broadband and high-repetition-rate coverage can be achieved by merging nonlinearly broadened electro-optic optical frequency combs with optical line-by-line spectrum shaping. Spectral bandwidth programming is implemented by iterative spectrum shaping and input power control of highly nonlinear stages, whereas repetition rate tuning is performed by modulation speed control in optical frequency combs. Herein, we implemented a programmable and tunable flat-topped supercontinuum with a maximum bandwidth and repetition rate of 55 nm at 10 dB and 50 GHz, respectively. To clarify the coherence of the supercontinuum during tuning and programming, we performed a phase-noise analysis. We proposed a remarkably modified self-heterodyne method to measure the phase noise of each mode precisely by filtering specific supercontinuum taps in a Mach–Zehnder interferometer. With this method, it has been proved that the single-sideband spectra in each mode are almost similar to that of the RF clock, indicating that our programmable and tunable supercontinuum generation process added minimal degradation to the phase noise properties. This study shows possibilities for generating hundreds of programmable and tunable flat-topped optical carriers with robustness and coherence.

## Introduction

Since the demonstration of the supercontinuum source based on an ultrashort pulse laser in the 1960s, it has received significant attention owing to its ultrabroadband and coherent characteristics^1^. As the spectral performance in terms of stability, bandwidth, and flatness has gradually improved, supercontinuum sources have been utilized in various fields, including optical communication system^2–4^, microwave photonics^5,6^, optical tomography^7,8^, and spectroscopy^9,10^. Because each application requires different characteristics of the supercontinuum sources^11^, the field of supercontinuum generation has been investigated to enhance the programmable performance of optical sources^12,13^. For instance, the required repetition rate for the combined spectroscopy application ranges from ~ 10 MHz to ~ 10 GHz^14^, whereas the calibration of astronomical spectrographs requires a repetition rate ranging from ~ 10 GHz to ~ 100 GHz^14^. Furthermore, there are some applications where the repetition rate needs to be adjusted even during use such as arbitrary waveform generation^15,16^.

Recent research efforts to implement supercontinuum generation with programmable and tunable characteristics include Kerr optical micro-combs^17,18^, mode-locked fiber lasers^19,20^, and electro-optic optical frequency combs (EO-OFC)^21,22^. Because the repetition rate and the spectral range of the Kerr optical micro-combs are determined by the material and the structure of the micro-resonator, attempts to program the repetition rates by either controlling the temperature^17^ or the applied electric field^18^ in the micro-comb have been made. Although Kerr optical micro-combs can offer attractive features, including a high repetition rate up to the THz and small size regime, the tuning range is narrow from the designed micro-resonator, and fine-tuning is difficult because of the step-like tuning range^12,17,18^.

Mode-locked fiber lasers have also been investigated for generating programmable and tunable supercontinuum sources^19,20^. A mode-locked soliton fiber laser with a tunable repetition rate was demonstrated using the opto-acoustic effect^19^. Temperature control of a Vernier filter, including two micro-ring resonators, has also been applied to generate tunable mode-locked fiber lasers^20^. Although mode-locked fiber lasers can generate an ultra-wideband supercontinuum with a broad bandwidth using a laser suitable for the desired wavelength range, the maximum repetition rate and tuning range are limited to several GHz and a few hundred MHz, respectively^12,19,20^. In addition, because the mode-locked fiber laser is tuned thermally or opto-acoustically, it is difficult to obtain a stable performance without additional feedback management.

The EO-OFC scheme overcomes the shortcomings of Kerr optical micro-combs and mode-locked fiber lasers in terms of flatness, robustness, programmability, and tunability. Additionally, it has the strength that the center wavelength can be adjusted easily and independently. The repetition rate can be adjusted continuously and precisely by adjusting the modulation speed of the intensity and phase modulation stages of the EO-OFC^21^. It is also possible to program the spectral bandwidth of supercontinuum optical sources by adjusting the light intensity into highly nonlinear stages^23^. With this scheme, there have been implementations of the programmable and tunable EO-OFC with a maximum repetition rate up to 18 GHz and tens of taps at 10 dB^21^. Although it showed the programmable and tunable possibilities of the EO-OFC scheme, the maximum repetition rate was limited to less than 20 GHz, and tens of taps were not suitable as supercontinuum sources.

In this study, we propose and demonstrate a programmable flat-top supercontinuum source based on the EO-OFC, including electro-optic intensity and phase-modulation schemes. When combined with the line-by-line pulse-shaping technique^24^, this solution provides the desired full programmability for the repetition rate, spectral bandwidth, and spectrum envelope. The programmability of the spectral bandwidth and the envelope is mainly performed by iterative optical line-by-line pulse shaping and optical power control into highly nonlinear stage, whereas the tunability of the repetition rate is implemented by modulation speed control in the EO-OFC. Herein, we demonstrate a flat-top supercontinuum source with a tunable repetition rate of 50 GHz and programmable spectral bandwidth of 55 nm at 10 dB. This enables programmable coverage of the S-, C-, and L-band regimes with the corresponding tunable line spacing for commercial optical switches and filters. To clarify the coherence of our supercontinuum sources during tuning and programming, we also performed a phase-noise analysis of the sources. We propose a modified self-heterodyne method to evaluate the phase noise of each mode of the supercontinuum source precisely. Using this platform, we proved that the single-sideband (SSB) spectra of the taps in every mode are very close to those of the RF clock, indicating that the phase noise properties of each mode are not significantly degraded by our supercontinuum generation process.

The remainder of this paper is organized as follows: The “Methods” are presented immediately after the “Introduction” section. Thereafter, the “Results” of the study are presented. Finally, the “Conclusions” are summarized.

## Methods

### Generating programmable and tunable flat-top supercontinuum

**Figure 1:**
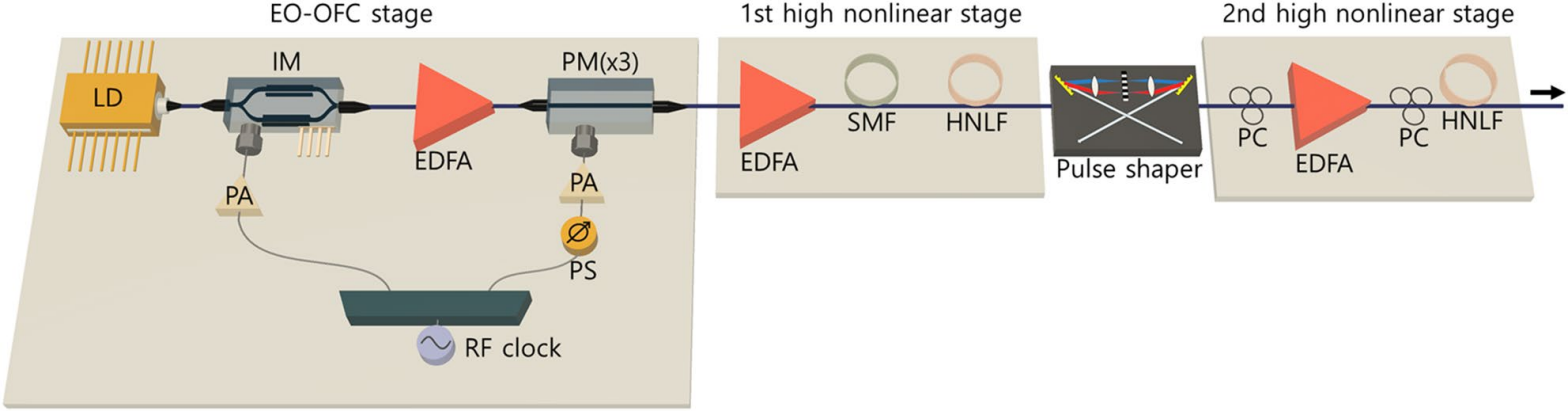
Schematic diagram of ultra-broadband flat-topped supercontinuum generator. *IM* optical intensity modulator, *PM* optical phase modulator, *EDFA* Erbium-doped fiber amplifier, *PA* power amplifier, *PS* phase shifter, *EDFA* Erbium-doped fiber amplifier, *SMF* single-mode fiber, *HNLF* highly nonlinear fiber, *PC* polarization controller.

To generate programmable and tunable flat-top supercontinuum sources with a high repetition rate and ultra-broad spectral bandwidth, we used an EO-OFC as a single-laser-fed multitap seed source. The EO-OFC has the advantage of allowing the repetition rate and center wavelength to be easily changed while maintaining a high degree of coherence and robustness. The schematic configuration of the EO-OFC is shown in Fig. 1, where a narrow-linewidth (< 0.1 kHz) continuous-wave (CW) laser output is sent through an electro-optic intensity modulator (IM) and three phase modulators (PM) set up in series^23^. The pulse shape of a CW laser is periodically carved through a properly biased IM, and the shaped pulses undergo time-to-frequency conversion in the cascaded PMs by applying a periodic time lens (linear chirp)^25^. An IM and three PMs are driven by an RF oscillator that generates a single frequency from 250 kHz to 50 GHz, where the repetition rate of the EO-OFC can be adjusted continuously by the driving frequency^26^.

**Figure 2:**
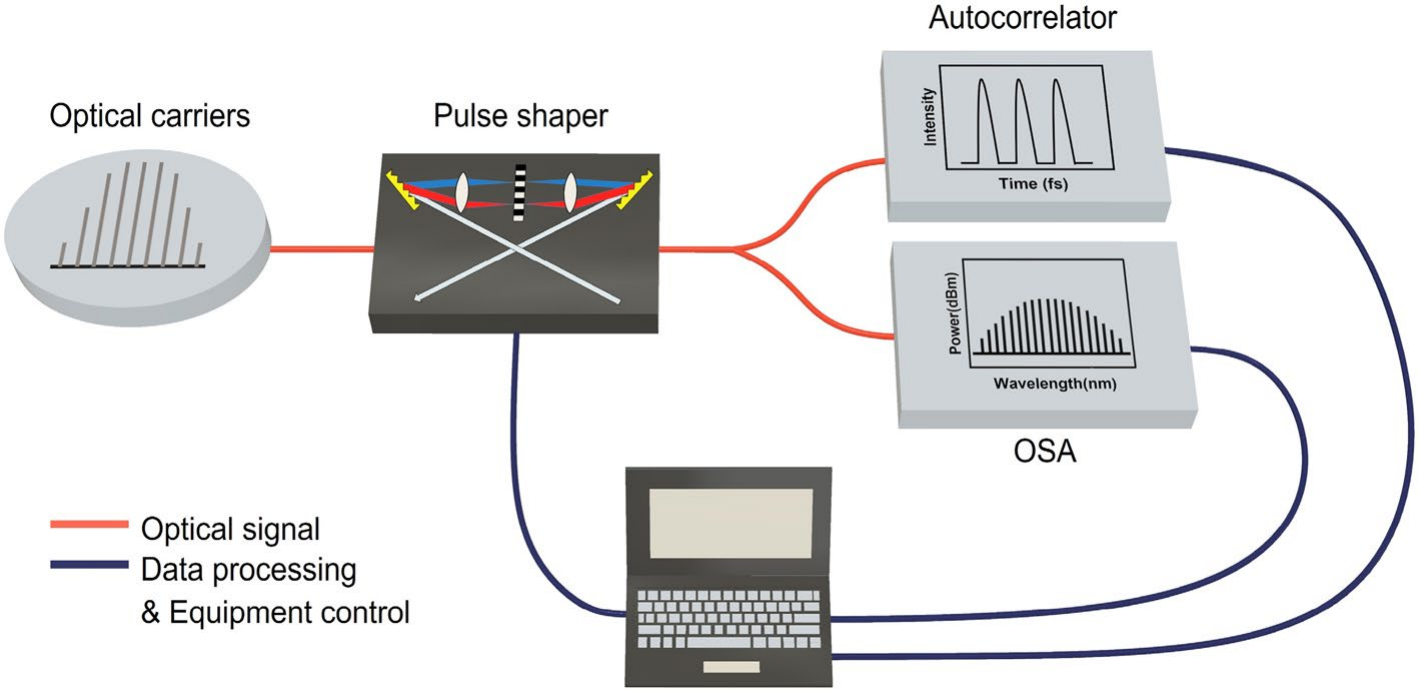
Schematic of programmable pulse phase and amplitude shaping. *OSA* optical spectrum analyzer.

In order to expand the spectrum bandwidth of the EO-OFC further before bandwidth-limited pulse shaping, we applied the first highly nonlinear stage. As shown in Fig. 1, after amplification through an Erbium doped fiber amplifier (EDFA), the EO-OFC passes through a dispersive linear single-mode fiber (SMF) spool to achieve near-zero dispersion by compensating for the dispersion of the EO-OFC stage. To enlarge the spectrum bandwidth, the dispersion-compensated EO-OFC is sent through a highly nonlinear fiber (HNLF), where spectral broadening occurs via self-phase modulation (SPM)^27,28^. The nonlinear phase shift (i.e., frequency shift) passing through a nonlinear medium is

1 $$ {\Phi } = - k_{0} n_{2} IL, $$

where *I* is the light intensity, *L* is the HNLF length, $k_{0}$ is the wavenumber ($k_{0} = 2\pi /\lambda_{0}$), and $n_{2}$ is the nonlinear refractive index. This equation mathematically indicates that the spectral bandwidth of supercontinuum sources can be adjusted by controlling the length of the nonlinear medium and light intensity. Because the loss also increases by lengthening the nonlinear medium, it was necessary to set the appropriate length of the nonlinear medium by considering the trade-off between spectral broadening and loss.The broadened EO-OFC via the first nonlinear stage was then programmed using a line-by-line optical pulse shaper with iterative spectral manipulation, where the amplitude and phase of the EO-OFC can be shaped precisely and simultaneously to maximize the flatness and bandwidth of the resulting supercontinuum spectrum. The line-by-line pulse shaping technique in the spectral regime enables the generation of the desired optical spectrum shape by a Fourier transform, which is called Fourier-transform optical spectrum shaping^27,29^. The programmable spectrum-shaping scheme is shown in Fig. 2, where the broadened EO-OFC from the first nonlinear stage enters the pulse shaper. The pulse shaper was then connected to an optical splitter with 50:50 coupling ratio to measure the spectrum and pulse profiles with an optical spectrum analyzer (OSA) and an autocorrelator.Since the difference between the transfer function and discrete seed spectrum data in the pulse shaper causes shaping errors, it is difficult to reach the desired shape with a single carving process. Furthermore, the liquid crystal in the pulse shaper has a weak coupling characteristic between amplitude and phase shaping. To obtain the desired target profile with a minimum error, we performed automatic monitoring with MATLAB to implement the iterative shaping^30^. After monitoring and calculating the difference between the measured and programmed target profiles, the line-by-line pulse shaper changed the amplitude and phase in turn. When the programmed taps are again entered into the pulse shaper, the difference level of the desired spectrum is re-calculated. The shaping process is completed when the phase and amplitude of each tap are designed in a user-defined manner.In order to implement an ultra-broadband flat-top supercontinuum, the input pulse to the second highly nonlinear stage must be quasi-super-Gaussian shape^23^. Although there have been several studies of supercontinuum generation with specific pulse shapes such as Gaussian^31–33^, parabolic^34^, and hyperbolic secant^35,36^ to improve the spectral flatness, the quasi-super-Gaussian shape proved the greatest flatness and bandwidth of supercontinuum generation^23^. The iterative amplitude shaping process apodizes and maintains the shape of the EO-OFC as quasi-super-Gaussian even when the repetition rate is changed. Iterative phase shaping also corrects the phase offset to maintain coherence during the programming and tuning processes.A flat-topped ultra-broadband supercontinuum can be generated by propagating the quasi-super-Gaussian-shaped EO-OFC to the second highly nonlinear stage, as shown in Fig. 1. To increase the spectrum bandwidth significantly, a highly nonlinear medium was pumped in the normal dispersion regime with a high power quasi-super-Gaussian-shaped pulse, resulting in strong SPM-induced nonlinear broadening^23^. As shown in Fig. 1, the combination of polarization controllers and an EDFA in the second highly nonlinear stage further improves the flatness and bandwidth of the supercontinuum spectrum via nonlinear polarization rotation reshaping^37^. As it acts as an intensity-dependent loss^37^, the sidelobes of optical pulses sent to the HNLF can be apodized by nonlinear polarization rotation. The spectral bandwidth of the supercontinuum spectrum can be easily and widely tuned by controlling the optical input power of the highly nonlinear medium as well as the quasi-super-Gaussian coefficient^23^.

### Phase noise measurement via an unprecedented remarkable method

**Figure 3:**
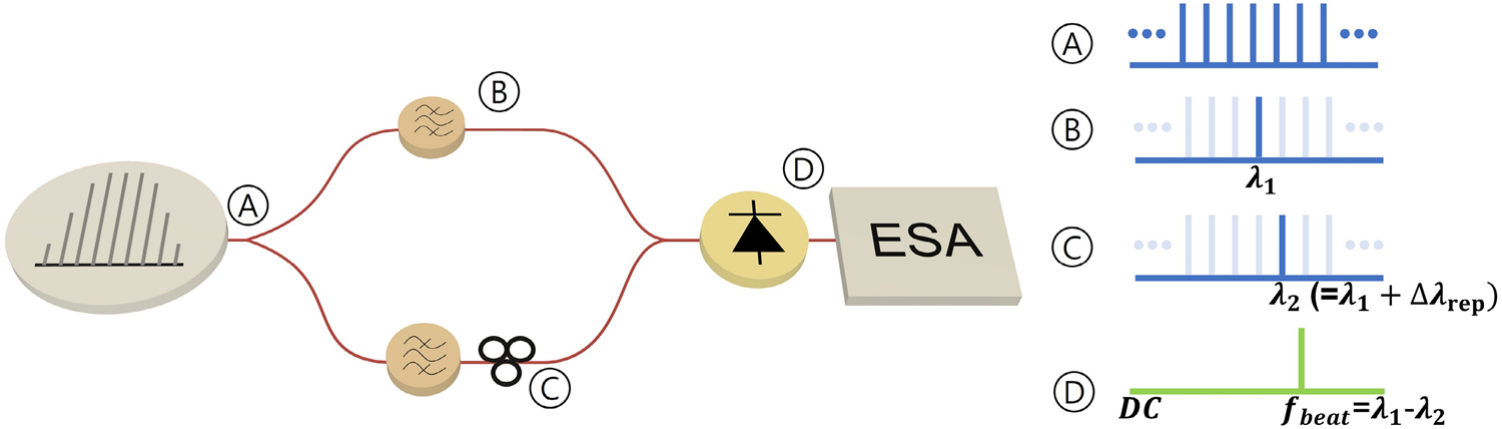
Schematic of phase noise measurement setup. *ESA* electrical spectrum analyzer.

Since the phase noise is a crucial factor in evaluating the coherence and frequency stability of optical sources, its characteristics are generally required for applications such as coherent optical communication, high-resolution spectroscopy, and optical precision metrology^38^. The optical linewidth measurement technique used to evaluate the phase noise is conventionally based on generating beat notes between a reference laser and the laser under test. The homodyne and heterodyne methods are representative techniques for measuring the optical linewidth, which use a Mach–Zehnder interferometer to convert the optical phase offsets of the laser into variations in light intensity^38,39^. The self-homodyne and self-heterodyne methods that use the laser under test as the reference laser have been promising for measuring the phase noise with a simple structure and the possibility of measuring the ultra-narrow linewidth^38^. However, the self-homodyne method is vulnerable to low frequency noise, and the self-heterodyne method requires an excessive length of delaying fiber and a frequency shift device such as an acousto-optic modulator^40^. When this measurement method is applied to measure the linewidth of supercontinuum sources, it also has an additional limitation in measuring the linewidth of each mode in the source.In order to measure the phase noise of each mode in the supercontinuum source precisely, we introduce a remarkable method by applying tunable filtering with pulse shapers. Since a supercontinuum source has multiple taps with a constant repetition rate, there is no need to use a frequency shifting device, as required by the self-heterodyne method, if two adjacent taps in a supercontinuum source are filtered to beat. Additionally, the scheme does not include an excessive length of delaying fiber, as the delay can be provided by the structural and material differences between the pulse shapers. It is suitable for evaluating the coherence of each mode by filtering specific spectral lines of the supercontinuum sources. The modified self-heterodyne configuration is schematically shown in Fig. 3. After dividing the supercontinuum into two paths through a 50:50 optical splitter, a specific carrier in both paths was filtered out through fiber-pigtailed programmable optical pulse shapers. The optical signal was detected by a photodiode (PD), and the power spectrum of the photocurrent fluctuations was measured using an electrical spectrum analyzer. In our experiment, we generated a programmable supercontinuum with a repetition rate of up to 50 GHz and measured the phase noise with a 50 GHz bandwidth PD. We filtered out two adjacent frequency components such that the frequency of the beat note did not exceed 50 GHz.

## Results

### Programmable and tunable flat-top supercontinuum generation

**Figure 4:**
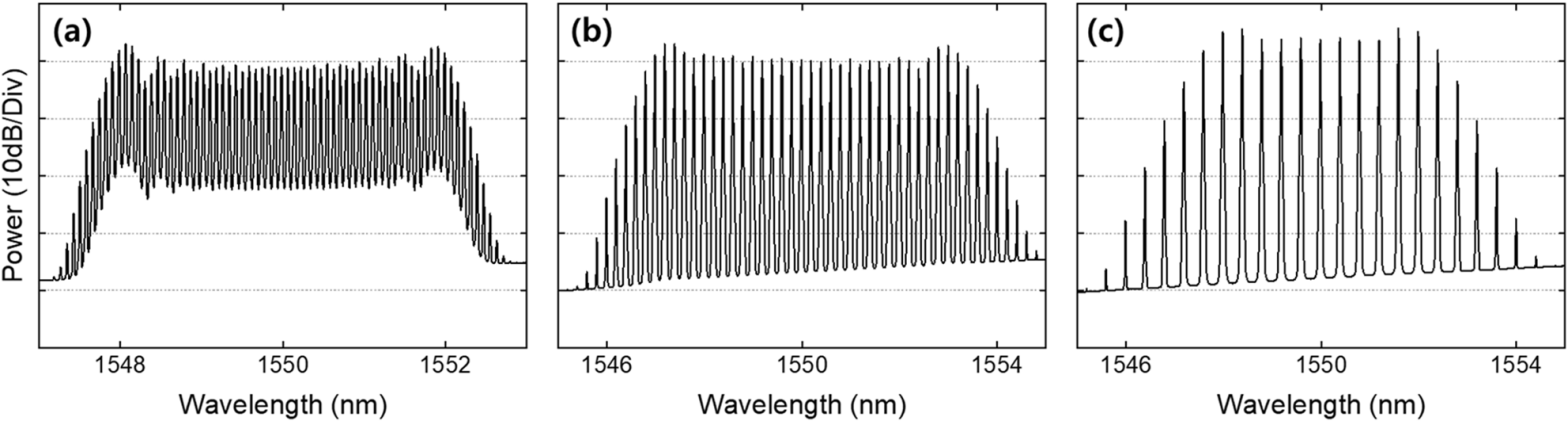
Experimentally measured optical spectrum of the EO-OFCs at repetition rates of (**a**) 10, (**b**) 25, and (**c**) 50 GHz.

**Figure 5:**
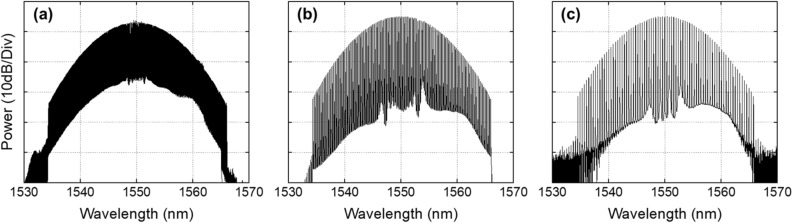
Spectral shaping results with a same quasi-super-Gaussian apodization at repetition rates of (**a**) 10, (**b**) 25, and (**c**) 50 GHz.

**Figure 6:**
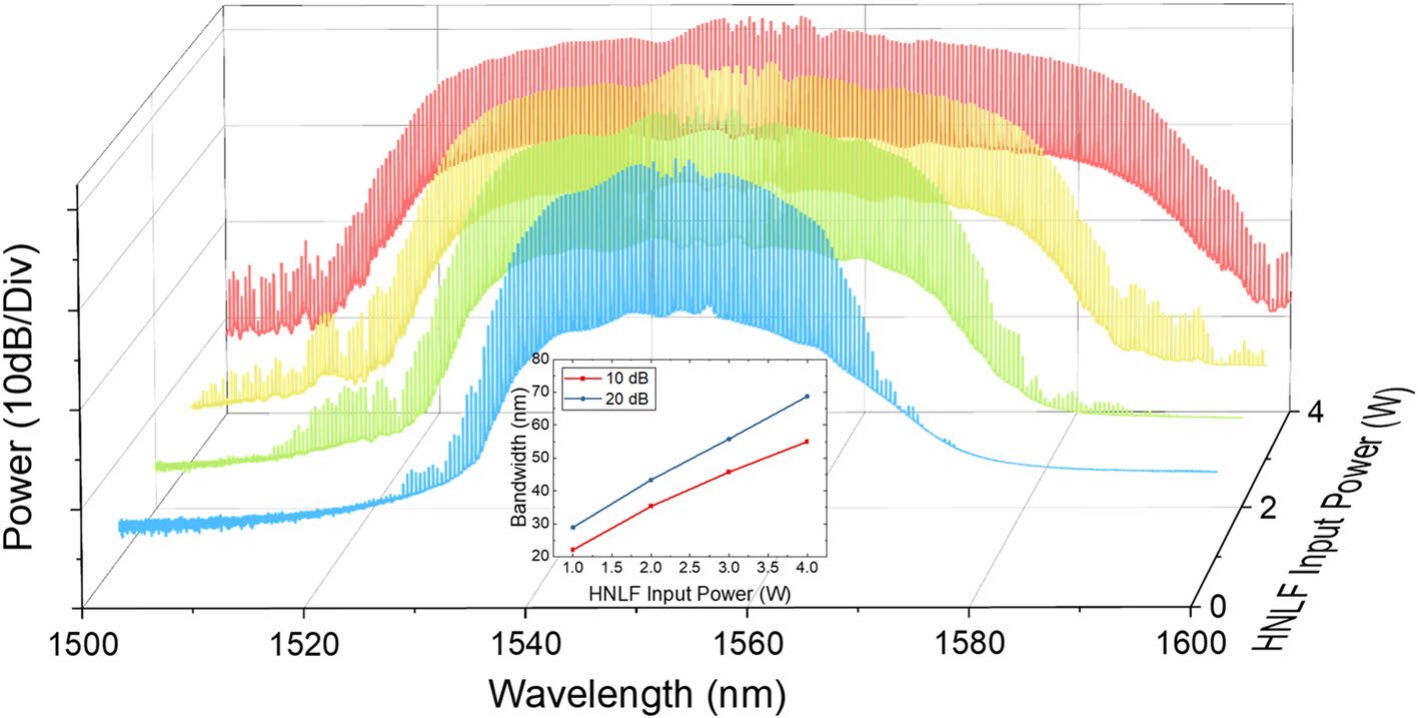
Experimentally measured optical spectrum of the bandwidth programmable supercontinuum, and inset shows 10 dB and 20 dB bandwidth according for HNLF input power.

In order to demonstrate the tunability of the repetition rate in the optical sources, we implemented EO-OFCs with repetition rates of 10, 25, and 50 GHz. Using the same configuration (see Fig. 1) but with different RF driving frequencies to the IM and PMs in the EO-OFCs, we implemented EO-OFCs with repetition rates of 10, 25, and 50 GHz, as shown in Fig. 4. The cascaded IM and three PMs were all driven by a tunable RF oscillator (Keysight, E8257D), which determined the desired repetition rate of the EO-OFC. The DC bias on the IM was set to generate a flat-top pulse, while the cusp of the PMs was aligned to match the peak of the pulse through phase shifters. Figure 4 shows the EO-OFC spectrum observed using an OSA with 0.01 nm resolution, where they lead to a flat envelope with 57, 36, and 15 taps at 10 dB bandwidth when the repetition rates are 10, 25, and 50 GHz, respectively. They show an optical signal-to-noise ratio above 35 dB with a similar shape and number of taps at different repetition rates, resulting in similar spectral characteristics after nonlinear broadening in the first highly nonlinear stage. In the nonlinear stage, the EO-OFCs with repetition rates of 10, 25, and 50 GHz were amplified to 26 dBm with an EDFA (LiComm, OFC-TCB-27AP) and the dispersion compensated with the SMF. The dispersion compensated EO-OFC was fed into 150 m of HNLF with a nonlinear coefficient of 11.5 W^−1^ km^−1^ and a dispersion of − 1.8 ps/nm/km, resulting in the spectrum bandwidth of ~ 20 nm at 10 dB for all the repetition rates.Thereafter, the nonlinearly broadened EO-OFC experiences iterative line-by-line amplitude and phase spectrum shaping. To generate optimal supercontinuum sources with minimized fluctuation in the flat-top regime, we adjusted the apodization profile to edge-cut super-Gaussian (i.e., quasi-super-Gaussian)^23^. To clarify the programming ability of our automatic iterative pulse shaping techniques, as shown in Fig. 5, we implemented the same apodization of the quasi-super-Gaussian while changing the repetition rate without any configuration changes. To maintain the apodization profile during repetition rate tuning, we programmed the iterative pulse shaping algorithm to sustain the apodization window by applying the changed apodization data to each pixel in the pulse shaper when the repetition rate change is recognized. Figure 5 shows the same apodization profile with 398, 159, and 79 taps above the edge at repetition rates of 10, 25, and 50 GHz, respectively. Because the apodization profile into the second highly nonlinear stage is dominant to the shape and bandwidth of supercontinuum sources, the apodization maintenance of the iterative pulse shaping enables the generation of the same supercontinuum envelope during repetition rate tuning without any structural change. The iterative line-by-line phase spectrum shaping also corrects the phase offset during the nonlinearly broadened EO-OFC generation and the tuning process.After amplitude and phase spectrum shaping, the quasi-super-Gaussian-shaped EO-OFC was propagated to the second highly nonlinear stage, as shown in Fig. 1. In order to implement the spectral bandwidth programming, we adjusted the optical input power to the highly nonlinear stage with an Erbium–Ytterbium co-doped optical fiber amplifier (PriTel, SP-LNHP-FA-37-IO-NMA), as shown in Fig. 6. After polarization control, the power-controlled spectrum was connected to 200 m of HNLF (− 2.22 ps/nm/km dispersion and 11.7 (W km)^−1^ nonlinear coefficient). As the optical power to the nonlinear stage increased, the spectrum bandwidth also increased linearly while maintaining flat-top properties. As shown in Fig. 6, when the input optical power is 1, 2, 3, and 4 W, the spectrum bandwidths at 10 dB are 22.1, 35.3, 45.7, and 55 nm, respectively. To improve and maintain the flatness of the programmable supercontinuum sources, we implemented further noise apodization between the adjacent taps. The linear relation between the input power and spectrum bandwidth is shown in the inset of Fig. 6, where the relation proves the possibility of additional enlarging of the spectrum bandwidth. As the input power of HNLF is limited by the maximum output power of the optical fiber amplifier, the spectrum bandwidth should be possible to increase further by using an optical fiber amplifier with the higher maximum output power.

### Amplitude and phase stability analysis of supercontinuum sources

**Figure 7:**
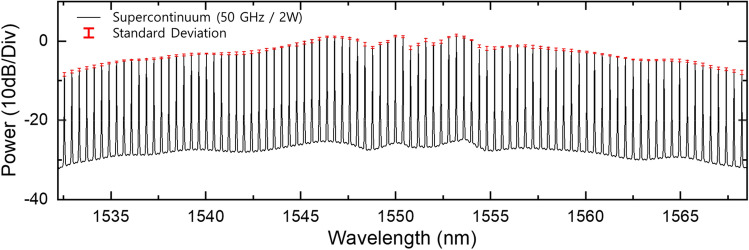
Experimentally measured supercontinuum spectrum and long-term stability measurement over 3 h. Black: supercontinuum spectra, red: standard deviation of supercontinuum.

**Figure 8:**
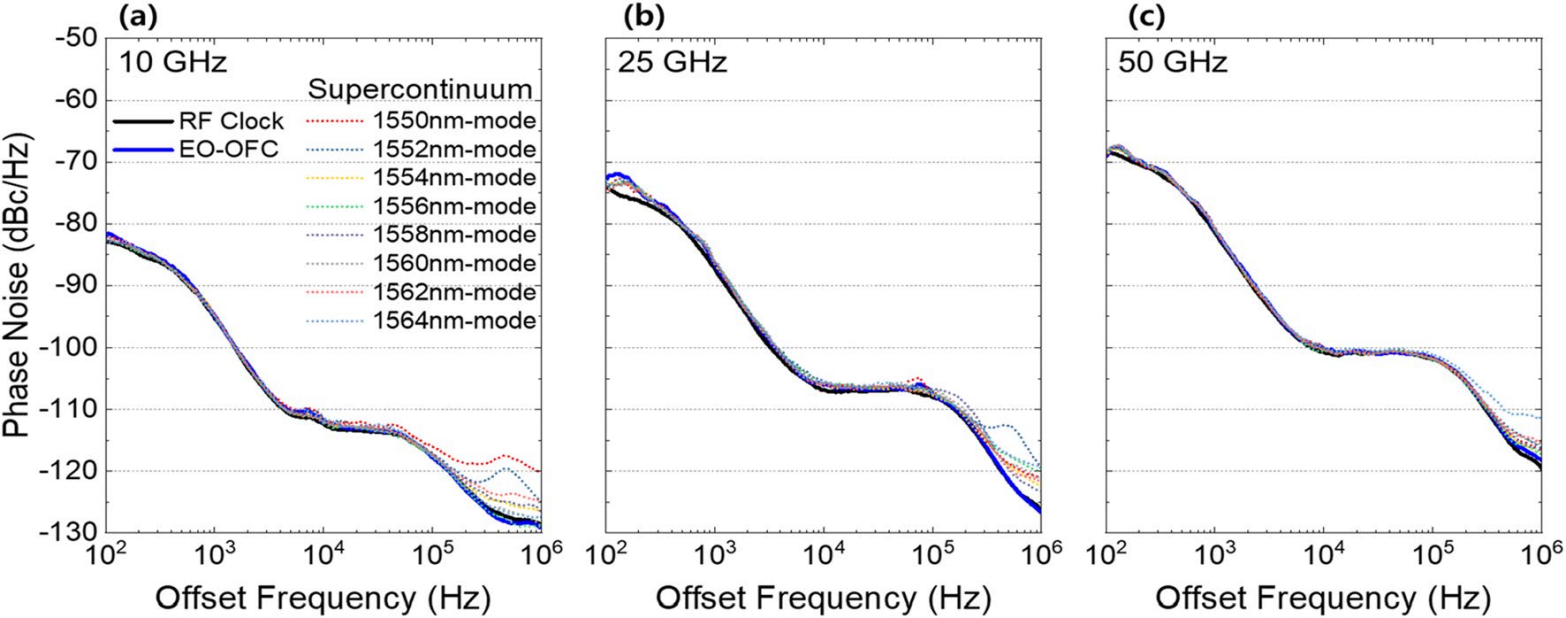
SSB RF spectrum-noise measurement at repetition rates of (**a**) 10, (**b**) 25, and (**c**) 50 GHz. The wavelength numbers of each mode show the shorter wavelength between the beaten taps.

In order to confirm the amplitude stability of the flat-top supercontinuum sources, we monitored the spectral trace with an OSA by letting it free run over a 3 h time window. In Fig. 7, the error bars overlaying the average value of the corresponding lines indicate the standard deviation of the amplitude fluctuations. We representatively measured the amplitude fluctuation when the supercontinuum source has a maximum spectral bandwidth and repetition rate of 35.3 nm at 10 dB and 50 GHz, respectively. The measured fluctuation result indicates a maximum standard deviation of 0.553 dB without any active stabilization, while they were acquired at 1 min intervals with a resolution of 0.01 nm.Additionally, we performed phase noise characterization to analyze the phase stability of supercontinuum sources. To clarify the coherence of the supercontinuum sources even during repetition rate programming, we measured the phase noise using our modified self-heterodyne method when the repetition rates of the optical carriers were 10, 25, and 50 GHz. Figure 8 shows the measured SSB spectra of the RF clock, EO-OFC, and supercontinuum sources for the three repetition rates. As shown in Fig. 8, the SSB spectra of the RF clock at 10 kHz offset are − 112.375, − 106.831, and − 100.703 dBc/Hz, and those of EO-OFC at 10 kHz offset are − 112.079, − 106.018, and − 100.118 dBc/Hz when the repetition rates are 10, 25, and 50 GHz, respectively. We also measured the SSB spectrum of each mode in supercontinuum sources to prove the coherence, where the phase noises of all modes were also very close to the RF clock and EO-OFC in each of the three different repetition rates. The SSB spectra were slightly deteriorated by phase noise accumulation during the supercontinuum generation processes at > 100 kHz offset, wherein the phase noise was mainly caused by the amplification of RF source and the bias point drift of electro-optic modulators.

## Conclusion

In summary, we demonstrated a programmable and tunable flat-topped supercontinuum laser source based on nonlinearly broadened EO-OFCs and an optical line-by-line pulse shaper. EO-OFCs composed of cascaded electro-optic IM and PMs are nonlinearly broadened by highly nonlinear stages. The phase and amplitude of each comb line were iteratively programmed with a spectrum shaper to maintain the shape of the EO-OFC as quasi-super-Gaussian even when the repetition rate is changed. Phase spectrum shaping also corrects the phase offset of the optical sources to maintain coherence during the programming and tuning processes. Spectral bandwidth programming of the supercontinuum source was implemented by iterative optical spectrum shaping and input optical power control of highly nonlinear stages. Repetition rate tuning was performed by controlling the modulation speed of the IM and PMs in the EO-OFCs. By applying programming and tuning techniques, we implemented a programmable and tunable flat-topped supercontinuum with a maximum spectral bandwidth and a repetition rate up to 55 nm bandwidth at 10 dB and 50 GHz, respectively. Additionally, we proposed an unprecedented modified self-heterodyne method to precisely measure the phase noise of each mode of supercontinuum sources. Through this platform, it has been proved that the single-sideband spectra in each mode are very close to the baseline of the RF clock, indicating that our supercontinuum generation process does not significantly degrade the phase noise properties. To the best of our knowledge, this study is the first to simultaneously achieve programmability and tunability in ultra-broad flat-top supercontinuum sources while maintaining robustness and coherence, which is compatible with the demanding requirements of modern communication and spectroscopy. This work also opens a new route for hyper-connected microwave photonic networks as flexible and versatile multiple optical carriers.
